# Age- and Sex-Specific Reference Values for Handgrip Strength Among Healthy Tunisian Adolescents

**DOI:** 10.3390/medicina61081383

**Published:** 2025-07-30

**Authors:** Souhail Bchini, Ismail Dergaa, Dhouha Moussaoui, Halil İbrahim Ceylan, Taoufik Selmi, Raul Ioan Muntean, Nadhir Hammami

**Affiliations:** 1 Research Unit (UR22JS01) “Sport Sciences, Health and Movement”, High Institute of Sport and Physical Education of Kef, University of Jendouba, Kef 7100, Tunisia; souhail.bchini@gmail.com (S.B.); phd.dergaa@gmail.com (I.D.); nadhir.hammami@issepkef.u-jendouba.tn (N.H.); 2 Higher Institute of Sport and Physical Education of Ksar Said, University of la Manouba, Manouba 2010, Tunisia; douhamoussaoui@yahoo.fr (D.M.); taoufikselmi72@gmail.com (T.S.); 3 Physical Activity, Sport and Health Research Unit, UR18JS01, National Observatory of Sport, Tunis 1003, Tunisia; 4 Physical Education and Sports Teaching Department, Faculty of Sports Sciences, Atatürk University, Erzurum 25240, Türkiye; 5 Department of Physical Education and Sport, Faculty of Law and Social Sciences, University “1 Decembrie 1918” of Alba Iulia, 510009 Alba Iulia, Romania

**Keywords:** adolescent health, dynamometry, musculoskeletal fitness, normative data, physical assessment, population health, reference standards, youth development

## Abstract

*Background and Objectives:* Handgrip strength represents a critical indicator of physical fitness and nutritional status in adolescents, yet population-specific reference values remain limited in developing countries. Understanding age- and sex-specific variations is crucial for accurate clinical assessment and effective health monitoring. The objective of this study was to establish comprehensive reference values for handgrip strength in healthy Tunisian adolescents aged 13–19 years and examine sex and age group differences in these measures. *Materials and Methods:* This cross-sectional study was conducted between September 2024 and June 2025, involving a sample of 950 participants (482 males, 468 females) aged 13–19 years from northwest Tunisia. Handgrip strength was measured using standardized dynamometry protocols for both hands. Anthropometric measurements included height, weight, and body mass index. Percentile curves were generated using the LMS method, and correlations between handgrip strength and anthropometric variables were analyzed using Pearson correlation coefficients. *Results:* Males demonstrated significantly higher handgrip strength than females from age 13 onward (13 years: *p* = 0.021; 14–19 years: *p* ≤ 0.001). Effect sizes for sex differences were consistently large across age groups (Cohen’s d range: 0.53–2.09 for the dominant hand). Mean dominant handgrip strength ranged from 25.60 ± 7.73 kg to 47.60 ± 12.45 kg in males and 21.90 ± 6.13 kg to 28.40 ± 4.74 kg in females across age groups. After adjusting for body mass, sex differences remained significant between groups (13 years: *p* = 0.014; d= 1.5; 14–19 years: *p* ≤ 0.001; d: 1.71–3.12). Strong positive correlations emerged between handgrip strength and height (males: r = 0.748, females: r = 0.601), body mass (males: r = 0.659, females: r = 0.601), and body mass index (BMI) (males: r = 0.391, females: r = 0.461). Body mass and height emerged as the strongest predictors of handgrip strength in both sexes, while BMI showed a smaller but still significant contribution. *Conclusions:* This study provides the first comprehensive age- and sex-specific reference values for handgrip strength in Tunisian adolescents. Healthcare providers can utilize these percentile charts for the clinical assessment and identification of musculoskeletal fitness deficits. The results suggest its use in educational and clinical contexts.

## 1. Introduction

Musculoskeletal fitness represents a fundamental component of overall health and physical development during adolescence, affecting approximately 1.2 billion adolescents worldwide [1]. The prevalence of musculoskeletal weakness in youth populations ranges from 15–30% globally, with significant implications for future health trajectories and chronic disease risk [2]. Poor muscular fitness during adolescence predicts an increased risk of mortality, cardiovascular disease, and metabolic disorders in adulthood [3,4]. The economic burden of musculoskeletal conditions in youth represents a significant healthcare challenge across developed and developing nations [1,5]. Demographic transitions in developing countries further amplify these concerns, as urbanization and lifestyle changes contribute to declining physical fitness levels among adolescent populations [6]. Given the importance of musculoskeletal fitness in adolescents, it is necessary to describe normative values and identify cut-off values below which health, growth, or development may be compromised or limited (health-referenced values) [7].

Handgrip strength assessment has emerged as a reliable and cost-effective marker of overall muscular fitness and health status in pediatric populations [8]. This measurement technique demonstrates strong correlations with total body strength, bone mineral density, and cardiovascular health markers across diverse adolescent populations [9,10]. The physiological basis of handgrip strength involves complex neuromuscular coordination, reflecting both muscle mass and neural drive capacity [8,11]. Clinical applications encompass nutritional status assessment, growth monitoring, and evaluation of sports performance [12,13]. The diagnostic utility extends to identifying children at risk for developmental delays, metabolic disorders, and future musculoskeletal complications [2,9,14].

The historical evolution of handgrip strength assessment reveals a progressive refinement of measurement protocols and the development of normative data across multiple populations [15,16]. Despite extensive research in developed countries, significant knowledge gaps persist regarding handgrip strength norms in developing nations, particularly among populations in North Africa [17,18]. Limited data availability from diverse ethnic and socioeconomic backgrounds restricts the generalizability of existing reference values [19]. Methodological inconsistencies across studies compromise the ability to establish universal standards and conduct meaningful cross-population comparisons [20,21]. Insufficient robust statistical analyses examining independent risk factors and confounding variables limit the clinical utility of current normative data [15,17]. The incomplete characterization of the relationships between handgrip strength and quality of life measures restricts the understanding of their functional significance [22]. A limited investigation of nutritional and dietary factors influencing strength development hinders the development of comprehensive health assessment protocols [12,13].

The absence of cost-effective screening tool validation in resource-limited settings prevents the widespread implementation of standardized assessment procedures [23]. Several international studies have developed reference values for adolescents [24,25]. In fact, variations in these handgrip strength levels suggest that norms developed for one country are not applicable to others, since the populations of different countries do not share homogeneous characteristics in social, economic, cultural, demographic, nutritional, and anthropometric aspects [26].

Handgrip strength is a widely accepted, dependable, and affordable measure of musculoskeletal fitness and general health in children and adolescents. It is closely linked to overall body strength, bone density, and indicators of cardiovascular health [27]. Research has also shown that handgrip strength can predict future health risks, such as cardiovascular disease, metabolic disorders, and even mortality [28]. Despite its importance in both clinical and public health contexts, there is a significant lack of population-specific reference data for handgrip strength in North Africa, especially among Tunisian adolescents. Most existing handgrip strength benchmarks come from studies conducted in Western, Asian, and South American countries [9], which may not accurately reflect the health profiles of Tunisian youth due to differences in genetics, nutrition, physical activity levels, and socioeconomic factors. This gap underscores the necessity for locally derived normative data to ensure that handgrip strength measurements are accurately interpreted in both medical and public health settings [29]. Moreover, global comparisons have shown considerable differences in handgrip strength values between populations, reinforcing the importance of developing region-specific standards to prevent misclassification and support effective, targeted health interventions [30]. In Tunisia, where rising urbanization, shifting dietary patterns, and reduced physical activity are increasingly affecting adolescent health, the assessment of musculoskeletal fitness through handgrip strength has become a crucial tool for monitoring and improving youth health outcomes.

The primary objective of this study is to establish the first comprehensive age- and sex-specific reference values for handgrip strength in healthy Tunisian adolescents aged 13–19 years, and to investigate sex- and age-related differences in handgrip strength performance. Secondary objectives are to explore the relationships between handgrip strength and key anthropometric variables (height, body mass, and BMI), and to compare the Tunisian handgrip strength profile with international data to highlight population-specific differences and contextualize our findings within the global context of musculoskeletal health.

We hypothesize that (1) Tunisian adolescents will show sex- and age-related differences in handgrip strength, with males exhibiting significantly higher strength values than females and strength increasing with age across both sexes; (2) handgrip strength will be strongly and positively correlated with height, body mass, and BMI, reflecting the influence of body size and composition on muscular strength; and (3) Tunisian adolescents will demonstrate lower handgrip strength values compared to their counterparts in high-income countries (e.g., Europe and North America), but higher than those in some South American populations, due to differences in nutritional status, physical activity patterns, and anthropometric characteristics.

## 2. Materials and Methods

### 2.1. Ethical Approval

This study received approval from the local research ethics committee of the High Institute of Sport and Physical Education at the University of Jendouba, El Kef, Tunisia (approval code: a18-2024, 28 January 2024). All procedures adhered to the Declaration of Helsinki for research involving human subjects [31]. Both participants and their parents provided written informed consent following a comprehensive explanation of study objectives and procedures. Participants retained the right to withdraw from the study at any time without penalty. It also complied with the ethical and procedural requirements for conducting sports medicine and exercise science research [32].

### 2.2. Study Design

This cross-sectional observational study was conducted between September 2024 and June 2025 across various regions of northwest Tunisia, including both urban and rural settings. Data collection took place in secondary schools and community centers, following approval from the Ministry of Education in Tunisia. A team of trained evaluators standardized all testing procedures to minimize measurement error and ensure data quality across multiple collection sites.

### 2.3. Sample Size Calculation

Sample size calculation was performed using the following formula for cross-sectional studies: *n* = (Z_1_ − α/_2_)^2^ × p (1 − p)/d^2^, where Z_1_ − α/_2_ represents the critical value for a two-tailed test at α = 0.05 (1.96), p represents the expected prevalence (0.5 for maximum variance), and d represents the desired precision (0.05). Based on previous studies by Cohen et al. [33] and Massy-Westropp et al. [34], with assumptions of a medium effect size (Cohen’s f = 0.25), a significance level of α = 0.05, and a target power of 80%, the analysis indicated a minimum sample size of 668 participants distributed across 14 groups (7 age groups × 2 sex categories).

### 2.4. Participants

The final sample comprised 950 adolescents aged 13–19 years (482 males and 468 females) recruited from secondary schools across northwest Tunisia. Inclusion criteria comprised: (i) age between 13–19 years; (ii) absence of known chronic illness (diabetes, cardiovascular disease, neuromuscular disorders); (iii) no physical disability affecting upper limb function; (iv) no ongoing treatment for acute illness; and (v) no history of recent injury or surgery involving the upper extremities. Exclusion criteria included incomplete data collection, failure to provide informed consent, and inability to complete testing procedures according to standardized protocols.

### 2.5. Experimental Procedures

Since this study involved standardized physical assessments, we ensured adherence to the highest standards in applying measurement protocols throughout the entire study, as highlighted by established guidelines for physical fitness testing in adolescent populations [35]. All testing sessions were conducted at the same time of day (between 09:00 and 11:00) to minimize potential bias and avoid any influence of circadian variations on the assessed variables [36,37].

Anthropometric Measurements: Body mass was measured using a digital scale (Harpenden Balance Scale, Holtain Ltd., Crosswell, UK) with participants wearing light clothing and no shoes. Standing height was assessed using a portable stadiometer (Portable Stadiometer, Holtain Ltd., Crosswell, UK) according to standard protocols [38]. BMI was calculated using the body mass-to-height ratio (kg/m^2^).

Handgrip Strength Assessment: Handgrip strength was measured using a calibrated Jamar^®^ manual dynamometer (Lafayette, LA, USA) with a resolution of 0.1 kg, following standardized protocols established by the American Society of Hand Therapists [39]. Participants stood upright with arms extended at their sides, shoulder adducted and neutrally rotated, elbow flexed at 90°, and forearm in neutral position. Three alternating measurements were taken from each hand with a one-minute rest interval between trials. Each maximal contraction was sustained for approximately 5 s, followed by complete relaxation. The highest value from each hand was selected for analysis. Handgrip strength values were normalized for body weight by dividing handgrip strength (kg) by participant body weight (kg).

### 2.6. Statistical Analysis

Statistical analysis was performed using SPSS software, Version 28.0 for Windows (IBM, New York, NY, USA). Normality was assessed using Kolmogorov–Smirnov tests for the overall sample and age–sex subgroups. Given the large sample size (*n* = 950) and subgroup sizes exceeding 30 participants, parametric tests remained appropriate despite mild deviations from normality, as per central limit theorem principles [40]. Independent *t*-tests compared handgrip strength between sexes, with Cohen’s d effect sizes calculated from sample means and pooled standard deviations. Cohen’s d was interpreted using the following thresholds: <0.20 (trivial); 0.20–0.60 (small); 0.60–1.20 (moderate); 1.20–2.0 (large); 2.0–4.0 (very large); and >4.0 (extremely large) [41]. Non-parametric Mann–Whitney U tests were applied to variables that showed significant normality violations in smaller subgroups [42]. Percentile values (P3, P5, P15, P25, P50, P75, P85, P90, P95, and P97) were derived using the Lambda-Mu-Sigma (LMS) method for modeling distribution curves as functions of age [43]. Comparisons between males and females at the same grade in anthropometric variables were made using Student’s unpaired *t*-tests. Comparisons between consecutive age groups (one group vs. the preceding) were performed using one-way ANOVA. When the ANOVA test was significant, a post hoc comparison analysis (Bonferroni) was used to determine differences between all age groups. Pearson correlation coefficients were used to assess the linear relationship between handgrip strength and anthropometric measures. Multiple regression analysis models were then used to determine the significant predictors of handgrip strength. Statistical significance was set at *p* < 0.05, with exact *p*-values reported throughout.

## 3. Results

### 3.1. Participant Characteristics

The study included 950 participants distributed across seven age groups: 13 years (*n* = 144), 14 years (*n* = 133), 15 years (*n* = 130), 16 years (*n* = 146), 17 years (*n* = 140), 18 years (*n* = 150), and 19 years (*n* = 107). A total of 890 participants (90.5%) were right-hand dominant, while 60 participants (9.5%) were left-hand dominant. The sex distribution remained balanced across all age groups, with males comprising 50.7% of the total sample, as shown in Table 1.

Anthropometric characteristics revealed significant sex differences in standing height, beginning at age 15 (*p* < 0.01), with males consistently demonstrating higher values than females. Body mass differed significantly between sexes (*p* < 0.01), with females showing higher values at younger ages. Body mass index differences achieved statistical significance only at age 14 (*p* < 0.01). All anthropometric characteristics showed significant age-related increases. Regression analysis revealed that age accounted for 68% of the variance in standing height among males (R^2^ = 0.68, *p* < 0.01) and 38% among females (R^2^ = 0.38, *p* < 0.01). For body mass, age explained 65% of the variance in males (R^2^ = 0.65, *p* < 0.01) and 48% in females (R^2^ = 0.48, *p* < 0.01) (Table 2).

### 3.2. Handgrip Strength Results

**Figure 1:**
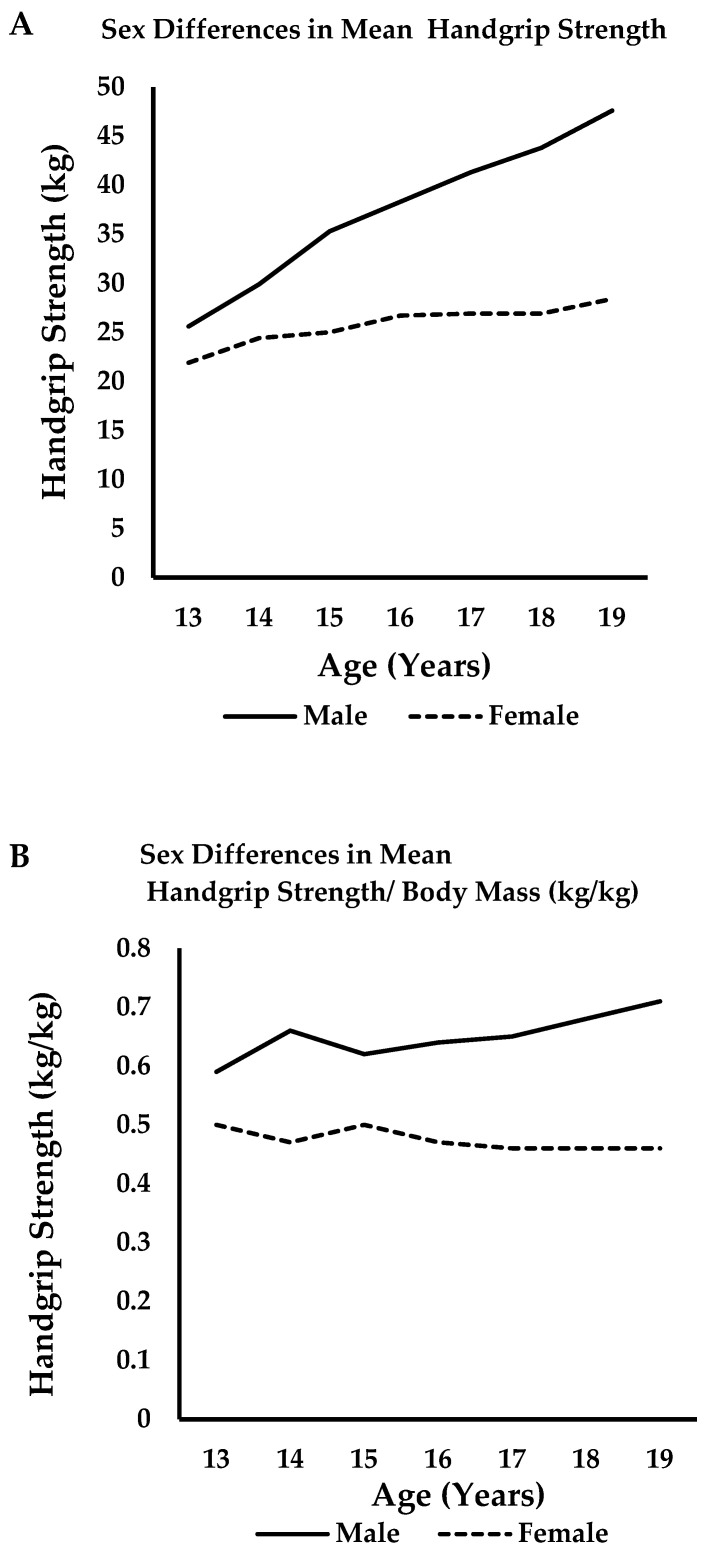
Sexual dimorphism in mean handgrip strength in males (solid line) and females (dashed line) aged 13–19 years: (**A**) unadjusted handgrip strength (kg) and (**B**) handgrip strength adjusted for body mass (kg/kg).

Males demonstrated significantly higher handgrip strength than females from age 13 onward (13 years: *p* = 0.021; 14–19 years: *p* ≤ 0.001). Effect sizes for sex differences were consistently large across age groups (Cohen’s d range: 0.53–2.09 for the dominant hand), indicating substantial and clinically meaningful differences that extend beyond statistical significance. Mean dominant hand strength in males ranged from 25.60 ± 7.73 kg at age 13 to 47.60 ± 12.45 kg at age 19. Corresponding values in females ranged from 21.90 ± 6.13 kg to 28.40 ± 4.74 kg across the same age span (Figure 1).

After adjusting for handgrip strength by body mass, statistically significant differences between sexes persisted across all age groups (13 years: *p* = 0.014; d = 1.5; 14–19 years: *p* ≤ 0.001; d: 1.71–3.12). Normalized strength values demonstrated progressive increases with age in males, while remaining relatively stable in females after the age of 15 (Table 3).

### 3.3. Percentile Distributions

**Figure 2:**
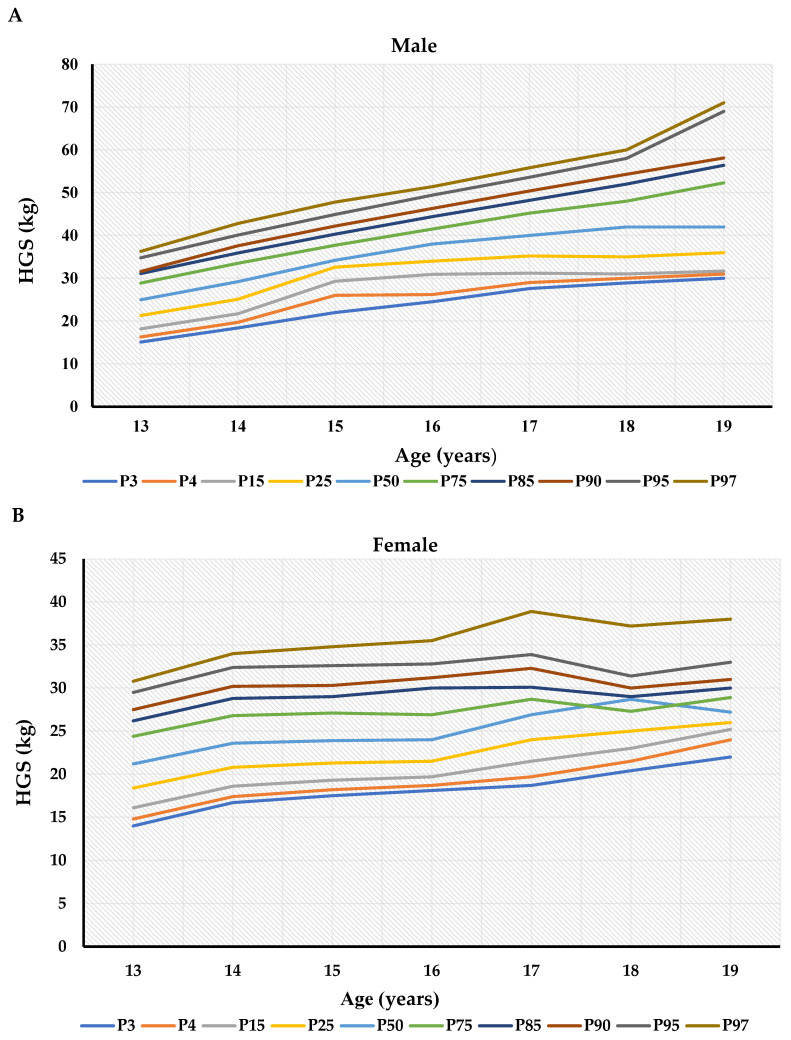
Percentile curves (P3, P5, P10, P25, P50, P75, P85, P90, P95, and P97) for dominant handgrip strength for males (**A**) and females (**B**) aged 13 to 19 years.

Comprehensive percentile tables were generated for both hands across all age groups and both sexes. Median values (P50) showed linear increases with advancing age in males, whereas females demonstrated plateau patterns after ages 16–17. The 95th percentile values for dominant hand strength reached 69 kg in 19-year-old males compared to 33 kg in age-matched females (Table 4). Figure 2 presents the dominant handgrip strength percentile curves for both sexes.

### 3.4. Correlations Between Anthropometric Variables and Handgrip Strength

While simple correlations showed positive associations between handgrip strength and anthropometric variables, these relationships likely reflect shared age-related growth patterns. Height showed the strongest correlations (males: r = 0.748; females: r = 0.601), suggesting its potential utility in developing predictive equations for clinical assessment when direct measurement is unavailable. Body mass correlations were r = 0.659 for males and r = 0.601 for females. BMI showed moderate correlations with handgrip strength (males: r = 0.391; females: r = 0.461) (Table 5).

### 3.5. Multiple Regression for Predicting Handgrip Strength in Healthy Tunisian Adolescents

Multiple regression analysis revealed that height, body mass, and BMI were the most significant predictors of handgrip strength. Height explained 49% of the variance in males (R^2^ = 0.49, *p* < 0.01) and 40% in females (R^2^ = 0.40, *p* < 0.01). Body mass accounted for 43% of the variance in males (R^2^ = 0.43, *p* < 0.01) and 36% in females (R^2^ = 0.36, *p* < 0.01). BMI explained 15% of the variance in males (R^2^ = 0.15, *p* < 0.01) and 21% in females (R^2^ = 0.21, *p* < 0.01) among both male and female adolescents (Table 6).

## 4. Discussion

### 4.1. Principal Findings Summary

This study established the first comprehensive age- and sex-specific reference values for handgrip strength in healthy Tunisian adolescents aged 13–19 years. Males demonstrated significantly higher handgrip strength than females from the age of 13 onward, with these differences persisting after adjustment for body mass. Strong positive correlations emerged between handgrip strength and anthropometric measures, with height showing the strongest associations. The percentile curves and reference tables provide essential tools for clinical assessment and population health monitoring in North African adolescent populations.

### 4.2. Sex Differences in Anthropometric and Handgrip Strength Development

The present study identified significant sex-related differences in anthropometric characteristics. From the age of 13 onward, male participants exhibited greater standing height compared to their female counterparts. This observation aligns with previous findings by Temfemo et al. [44], who reported similar sex disparities in standing height among healthy adolescents. Regarding body mass, statistical analysis revealed significantly higher values in males starting at the age of 14. These results are partially consistent with those documented by Beenakker et al. [45] and Temfemo et al. [44]. Furthermore, body mass index (BMI) demonstrated a significant increase with age, which may be attributed to the physiological processes of growth and maturation, as previously described by Rogol et al. [46]. All anthropometric variables, standing height, body mass, and BMI, increased significantly with age. This trend is consistent with the physiological processes of growth and biological maturation during adolescence [46].

The emergence of significant sexual dimorphism in handgrip strength from the age of 13 onward aligns with established patterns observed in European populations [3,4]. These findings support biological mechanisms involving hormonal changes during puberty, particularly increased testosterone production in males, which leads to enhanced muscle mass and strength development [47,48]. Similar patterns have been documented in French adolescents, where males demonstrated 15–20% higher grip strength than females across corresponding age groups [49]. The consistency across diverse populations suggests that universal developmental patterns are influenced by pubertal hormonal changes rather than cultural or environmental factors [2,47]. Colombian research revealed similar sexual dimorphism patterns, although absolute values were lower than those observed in our Tunisian sample [50]. Portuguese studies have demonstrated comparable developmental trajectories, with males maintaining superior strength throughout adolescence [51]. The biological basis for these differences involves complex interactions among growth hormone, insulin-like growth factor-1, and sex hormones during pubertal development [47,48,52].

### 4.3. Anthropometric Relationships and Body Size Influences

The strong correlations between handgrip strength and anthropometric variables, particularly height (r = 0.748 in males and r = 0.601 in females), support established biomechanical principles that link body size to muscular force production [53]. These relationships reflect the fundamental influence of lever arm length, muscle cross-sectional area, and overall body mass on force generation capacity [38,53]. Similar correlation patterns have been documented across multiple populations, including Dutch children and American adolescents [33,34,54]. When comparing our findings to international data, Chilean and Colombian adolescents demonstrated lower absolute handgrip strength values, which can be attributed to differences in average body size and hand dimensions [24]. Adjustment for body mass significantly reduced these population differences, highlighting the importance of normalization procedures when conducting cross-cultural comparisons [17,18]. The relationship between handgrip strength and BMI (r = 0.391–0.461) suggests a moderate influence of body composition on strength performance [53,54].

The multiple regression analysis conducted in this study demonstrates that anthropometric variables significantly predict HGS in healthy Tunisian adolescents, with standing height, body mass, and BMI identified as the most influential predictors. These findings are consistent with previous research indicating that body size and composition are closely associated with muscular strength during growth and development [55,56]. The strong relationship between HGS and anthropometric parameters underscores the importance of considering somatic maturation when assessing musculoskeletal fitness in adolescent populations [57].

The observed sex differences in R^2^ values across all three anthropometric predictors suggest that the relationship between body size and strength development follows distinct trajectories in male and female adolescents. Males exhibited higher predictive power for height and body mass, whereas females showed relatively stronger associations with BMI. This pattern may indicate that absolute growth parameters play a more dominant role in musculoskeletal development among boys, while girls may be more influenced by body composition and fat distribution. These differences are likely attributable to the divergent timing, tempo, and hormonal regulation of pubertal maturation between the sexes [58,59].

### 4.4. Clinical and Practical Applications

These reference values provide healthcare providers with essential tools for assessing muscular fitness and identifying adolescents at risk for developmental delays or health complications [8,9]. Clinical applications include the evaluation of nutritional status, where handgrip strength serves as a sensitive indicator of protein-energy malnutrition and micronutrient deficiencies [12,13]. Sports medicine practitioners can utilize these norms for talent identification, performance monitoring, and injury risk assessment [60]. Educational institutions benefit from standardized assessment protocols for evaluating physical education and fitness tracking [35]. The percentile approach enables the identification of adolescents below the 10th percentile, who may require additional assessment or intervention [8,22]. Healthcare systems can implement these standards to support population health surveillance and informed resource allocation decisions [1,23]. The strong correlation with height suggests potential for developing predictive equations for clinical use when direct measurement is not feasible [38,53]. Public health applications include monitoring secular trends in adolescent fitness and evaluating the effectiveness of interventions [1,6].

### 4.5. International Comparisons and Population Variations

Our results indicate that Tunisian adolescents exhibit handgrip strength values intermediate to those reported in European and South American populations [17,19], suggesting moderate musculoskeletal fitness relative to global standards. These differences are likely influenced by variations in body composition, nutritional status, physical activity levels, and socioeconomic conditions—key factors known to affect muscular development during adolescence [6,18]. When compared to normative data from high-income countries such as the United States, Tunisian adolescents demonstrated approximately 10–15% lower absolute handgrip strength values [33,34]. This disparity may reflect differences in average body size, dietary intake, and lifestyle behaviors, such as reduced participation in structured physical activity or sports, among North African youth. Notably, when handgrip strength was normalized for body mass, the inter-regional differences were reduced, highlighting the importance of adjusting for anthropometric variables when interpreting cross-population comparisons. Despite these variations, our findings support the global applicability of handgrip strength as a valid and reliable indicator of overall muscular fitness in adolescent populations [15,17,19]. The consistent age- and sex-related developmental trajectories observed in our sample align with patterns reported in other regions, reinforcing the biological basis of strength gains during puberty, particularly under the influence of sex hormones such as testosterone in males [47,48]. However, our results underscore the necessity of population-specific reference values. Applying norms derived from other regions may lead to misclassification and the implementation of inappropriate health interventions. As the first comprehensive study of its kind, this research provides age- and sex-specific reference values for handgrip strength in Tunisian adolescents aged 13–19 years, addressing a critical gap in the literature. These data not only enhance individual health assessments but also support public health initiatives aimed at monitoring trends in adolescent physical development and designing targeted interventions to improve musculoskeletal health in North Africa. Furthermore, they contribute to global health databases, enabling meaningful cross-population comparisons and informing evidence-based international health policies. These reference values fill a critical gap for North African populations and provide foundation data for regional health monitoring [1,23].

### 4.6. Methodological Considerations and Strengths

The use of standardized Jamar dynamometry protocols ensures consistency with international research and enables valid comparisons across populations [39]. The large sample size (*n* = 950) provides adequate power for percentile generation and subgroup analyses across seven age groups. The LMS method for generating percentile curves represents the gold standard approach for developing reference values in pediatric populations [43]. A comprehensive anthropometric assessment enables the adjustment for body size differences and facilitates correlation analyses [38].

Quality control measures included evaluator training, equipment calibration, and standardized testing conditions to minimize measurement error [35,39]. The time-of-day standardization addresses potential circadian influences on strength performance [36,37]. The broad geographic recruitment across northwest Tunisia enhances the representativeness of the regional adolescent population.

### 4.7. Study Limitations

The cross-sectional design prevents examination of individual developmental trajectories and limits causal inferences regarding factors influencing strength development [61]. Regional specificity to northwest Tunisia may limit generalizability to other North African populations with different genetic, nutritional, or socioeconomic characteristics [18,19]. The absence of physical activity and nutritional status assessments prevents the examination of lifestyle factors that potentially influence handgrip strength [6,12]. Pubertal stage assessment was not included, which limited the understanding of maturational influences on strength development patterns [47,48]. Additionally, BMI was used as a general indicator of body composition; however, it is only a very rough proxy and does not distinguish between fat mass and muscle/lean mass. Self-reported health status may have introduced selection bias, as participants with undiagnosed conditions could have been included in the reference sample. The study period spanning the academic year may have introduced seasonal variations in physical activity and strength performance [6]. Future longitudinal studies are needed to validate these cross-sectional findings and examine individual developmental patterns [61]. Subgroup sample sizes of approximately 70 participants per age–sex combination, while adequate for statistical analysis, may limit the precision of extreme percentiles (P3, P97) for clinical decision-making. Larger samples would enhance the reliability of tail percentiles, particularly important for identifying adolescents requiring clinical intervention [62].

### 4.8. Future Research Directions and Clinical Translation

Longitudinal studies should track individual adolescents over multiple years to characterize personal development trajectories and identify factors associated with optimal strength development [61]. Investigation of predictive relationships between adolescent handgrip strength and adult health outcomes would enhance clinical utility [3,9]. The development of population-specific prediction equations incorporating anthropometric variables could facilitate clinical application [38,53]. Research examining cultural and lifestyle factors influencing strength development would inform targeted interventions [6,18]. Validation studies in other North African populations would enhance the regional applicability of these reference values [19,23]. Cost-effectiveness analyses of handgrip strength screening programs would support healthcare policy decisions [1,8]. Integration with other fitness measures could provide comprehensive adolescent health assessment protocols [22,35]. Investigating the relationships between handgrip strength and quality of life measures would clarify the functional significance [9,22].

## 5. Conclusions

This study provides the first comprehensive age- and sex-specific reference values for handgrip strength in healthy Tunisian adolescents aged 13–19 years, addressing a critical knowledge gap in North African populations. The establishment of percentile curves using the LMS method enables precise classification of individual performance relative to population norms. Healthcare providers should utilize these reference standards for clinical assessment, evaluation of nutritional status, and identification of adolescents who require additional investigation or intervention. Educational institutions can implement these norms for physical education assessment, fitness monitoring, and sports talent identification programs.

## Figures and Tables

**Table 1:** Distribution of participants by age group (13–19 years).

Age (Years)	Total (*n*)	Male (*n*)	Female (*n*)
13	144	75	69
14	133	68	65
15	130	60	70
16	146	76	70
17	140	75	65
18	150	78	72
19	107	50	57
Total	950	482	468

**Table 2:** Anthropometric variables in healthy Tunisian adolescents: mean ± SD by age and sex.

		Age						
Variables	Sex	13 Years	14 Years	15 Years	16 Years	17 Years	18 Years	19 Years
Height (m)	Male	1.57 ± 0.04	1.60 ± 0.09 ^f^	1.68 ± 0.07 ^a^	1.71 ± 0.05	1.73 ± 0.06	1.75 ± 0.05	1.75 ± 0.05
	Female	1.53 ± 0.05 ^b^	1.59 ± 0.05	1.55 ± 0.07 ^b^	1.63 ± 0.07 ^b^	1.64 ± 0.05 ^b^	1.64 ± 0.05 ^b^	1.65 ± 0.05 ^b^
Body mass (kg)	Male	43.10 ± 7.3	45.00 ± 7.2 ^e^	56.73 ± 8.07 ^a,e^	59.15 ± 7.47 ^e^	63.04 ± 8.96	63.63 ± 7.23	66.60 ± 11.38 ^e^
	Female	44.00 ± 6.91	52.00 ± 13.9	49.26 ± 0.06	53.77 ± 0.08	58.20 ± 0.07 ^b^	58.90 ± 6.42 ^b^	62.14 ± 9.11
BMI (kg/m^2^)	Male	18.27 ± 2.2	17.41 ± 1.56 ^b^	20.03 ± 2.28 ^a^	20.04 ± 2.16	20.80 ± 2.36	20.24 ± 1.93	21.40 ± 3.12
	Female	17.70 ± 2.04	20.80 ± 5.37	19.20 ± 2.01	20.10 ± 2.42	21.50 ± 2.53	21.00 ± 2.32	22.80 ± 3.08

^a^ Male: age vs. previous age: *p* < 0.01. ^b^ Significant difference between males and females in the same age group (*p* < 0.01). ^e^ Significant difference between males and females in the same age group (*p* < 0.05). ^f^ Male: age vs. previous age: *p* < 0.05.

**Table 3:** Mean ± standard deviation, mean difference, 95% confidence interval, and Cohen’s d effect sizes of right and left handgrip strength by sex and age group, unadjusted and adjusted for body mass (kg). Effect sizes calculated using pooled standard deviations.

	Right Hand (kg)						Right Hand (kg/kg)					
Age	Male	Female	Mean Diff	95% CI	*p*	Cohen’s d	Male	Female	Mean Diff	95% CI	*p*	Cohen’s d
13	25.60 ± 7.73	21.90 ± 6.13	3.7	(1.8, 5.2)	0.021	0.53	0.59 ± 0.02	0.50 ± 0.01	0.09	(0.03, 0.12)	0.014	1.50
14	29.90 ± 8.81	24.40 ± 6.53	5.5	(2.7, 8.2)	0.012	0.71	0.66 ± 0.08	0.47 ± 0.07	0.19	(0.14, 0.24)	≤0.001	2.71
15	35.30 ± 8.15	25.00 ± 6.18	10.5	(6.8, 13.2)	≤0.001	1.44	0.62 ± 0.10	0.50 ± 0.02	0.12	(0.09, 0.18)	0.001	1.71
16	38.30 ± 9.53	25.40 ± 6.33	12.9	(8.4, 15.2)	≤0.001	1.58	0.64 ± 0.09	0.47 ± 0.06	0.17	(0.13, 0.24)	≤0.001	2.12
17	41.30 ± 10.40	26.70 ± 6.16	14.6	(11.1, 17.2)	≤0.001	1.68	0.65 ± 0.08	0.46 ± 0.06	0.19	(0.15, 0.26)	≤0.001	2.71
18	43.80 ± 12.19	26.90 ± 5.11	16.9	(13.8, 19.2)	≤0.001	1.78	0.68 ± 0.09	0.46 ± 0.02	0.22	(0.18, 0.29)	≤0.001	3.66
19	47.60 ± 12.45	28.40 ± 4.74	19.2	(14.8, 21.2)	≤0.001	2.09	0.71 ± 0.10	0.46 ± 0.10	0.25	(0.20, 0.29)	≤0.001	3.12
	Left hand (kg)						Left hand (kg/kg)					
13	24.80± 8.07	21.40 ± 5.54	3.4	(1.6, 5.1)	0.032	0.49	0.57 ± 0.10	0.49 ± 0.06	0.08	(0.02, 0.11)	0.015	1.14
14	29.60 ± 8.86	22.90 ± 5.93	6.7	(3.8, 9.7)	0.011	0.88	0.65 ± 0.10	0.44 ± 0.10	0.21	(0.16, 0.25)	≤0.001	2.10
15	33.20 ± 9.32	23.90 ± 5.93	9.3	(6.4, 12.1)	≤0.001	1.21	0.58 ± 0.08	0.48 ± 0.05	0.10	(0.06, 0.17)	0.001	1.66
16	36.50 ± 9.70	23.90 ± 5.93	12.6	(8.1, 14.9)	≤0.001	1.55	0.62 ± 0.10	0.44 ± 0.10	0.18	(0.13, 0.25)	≤0.001	1.80
17	39.40 ± 10.20	24.80 ± 5.80	14.6	(10.9, 17.1)	≤0.001	1.73	0.62 ± 0.11	0.43± 0.10	0.19	(0.16, 0.26)	≤0.001	1.90
18	42.10 ± 12.57	26.60 ± 6.92	15.5	(12.9, 18.8)	≤0.001	1.51	0.66 ± 0.13	0.45 ± 0.10	0.21	(0.17, 0.29)	≤0.001	2.10
19	46.40 ± 15.29	27.00 ± 5.37	19.4	(15.4, 22.6)	≤0.001	1.74	0.70 ± 0.14	0.43 ± 0.11	0.27	(0.20, 0.31)	≤0.001	2.70

**Table 4:** Percentile distribution of handgrip strength (HGS) in both the right and left hands for adolescents of both sexes, according to age.

	Handgrip Strength (kg)													
Sex	Males							Females						
Age (Years)	13	14	15	16	17	18	19	13	14	15	16	17	18	19
Right hand														
P3	15.10	18.40	22.00	24.50	27.60	28.90	30.00	14.00	16.70	17.50	18.10	18.70	20.40	22.00
P4	16.30	19.70	26.00	26.20	29.00	30.00	31.00	14.80	17.40	18.20	18.70	19.70	21.50	24.00
P15	18.20	21.70	29.30	30.90	31.20	31.00	31.70	16.10	18.60	19.30	19.70	21.50	23.00	25.20
P25	21.30	25.10	32.60	34.00	35.20	35.00	36.00	18.40	20.80	21.30	21.50	24.00	25.00	26.00
P50	25.00	29.20	34.20	38.00	40.00	42.00	42.00	21.20	23.60	23.90	24.00	26.90	26.00	27.20
P75	28.90	33.50	37.70	41.50	45.20	48.00	52.30	24.40	26.80	27.10	26.90	28.70	27.30	28.90
P85	31.10	35.90	40.30	44.40	48.20	52.00	56.40	26.20	28.80	29.00	30.00	30.10	29.00	30.00
P90	32.60	37.60	42.20	46.30	50.40	54.30	58.10	27.50	30.20	30.30	31.20	32.30	30.00	31.00
P95	34.80	40.10	44.90	49.40	53.60	58.00	69.00	29.50	32.40	32.60	32.80	33.90	31.40	33.00
P97	36.30	42.80	47.80	51.40	55.80	60.00	71.00	30.80	34.00	34.80	35.50	36.90	37.20	38.00
Left hand														
P3	14.60	18.10	21.30	24.10	26.80	26.50	28.60	14.50	15.80	16.60	16.80	17.80	18.80	19.00
P4	15.70	19.20	22.50	25.30	28.00	28.00	30.00	15.20	16.50	17.20	17.50	18.50	19.50	21.00
P15	17.30	21.00	24.30	27.10	29.80	28.90	31.00	16.20	17.60	18.30	18.60	19.50	20.00	23.00
P25	20.30	24.30	27.70	30.60	33.30	33.90	35.00	18.20	19.60	20.40	20.60	21.50	22.50	23.60
P50	23.90	28.30	32.00	35.00	37.80	40.50	43.90	20.70	22.10	23.00	23.20	24.10	25.00	28.00
P75	28.00	32.80	36.80	39.90	42.90	48.00	50.40	23.60	25.00	26.00	26.10	27.00	29.90	28.90
P85	30.30	35.40	39.60	42.90	46.00	51.10	54.50	25.30	26.90	27.80	27.90	28.70	31.80	29.00
P90	31.90	37.20	41.60	45.00	48.30	53.70	57.10	26.60	28.20	29.10	29.20	30.00	32.00	31.00
P95	34.50	40.10	44.70	48.40	51.80	56.00	67.00	28.60	30.30	31.20	31.30	31.90	35.80	32.00
P97	36.20	42.10	46.90	50.70	54.30	58.10	69.00	29.90	31.70	32.60	32.70	33.30	35.80	36.70

**Table 5:** Correlation coefficients between handgrip strength and anthropometric variables.

Variables	Sex	Dominant Handgrip Strength
Height (m)	Male	0.748 **
	Female	0.601 **
Body mass (kg)	Male	0.659 **
	Female	0.601 **
BMI (kg/m^2^)	Male	0.391 **
	Female	0.461 **

** *p* < 0.01. The *p*-values were calculated using Pearson’s correlation analysis.

**Table 6:** Regression analysis of anthropometric characteristics and handgrip strength.

Variables	Sex	R^2^	*p* Value
Height (m)	Male	0.49	*p* < 0.01
	Female	0.40	*p* < 0.01
Body mass (kg)	Male	0.43	*p* < 0.01
	Female	0.36	*p* < 0.01
BMI (kg/m^2^)	Male	0.15	*p* < 0.01
	Female	0.21	*p* < 0.01

## Data Availability

The data that support the findings of this study are available from the corresponding author upon reasonable request.
